# "The Hospital" Medical Book Supplement—No. XXX

**Published:** 1910-06-18

**Authors:** 


### "THE HOSPITAL"
### MEDICAL BOOK SUPPLEMENT—No. XXX

CONTENTS.

| | Page |
|---|---|
| **Medicine—** | |
| The Rearing of Children: A Practical Handbook for Mothers | 357 |
| Insanity in Everyday Practice | 357 |
| Variations in Susceptibility of Guinea-pigs to Diphtheria Toxin | 357 |
| An Index of Symptoms | 358 |
| The Action of an Active Principle from Apocynum | 358 |
| **Surgery—** | |
| Four Common Surgical Operations in India | 358 |
| Diseases of the Colon and their Surgical Treatment | 359 |
| **Tropical Medicine—** | |
| Lessons on Elementary Hygiene and Sanitation, with Special Reference to the Tropics | 359 |
| A Guide to the Preservation of Health in West Africa | 359 |
| Health, Progress, and Administration in the West Indies | 359 |
| **Miscellaneous—** | |
| The History of St. George's Hospital | 360 |
| City of London Year Book and Civic Directory for 1910 | 360 |
| History of the Human Body | 360 |

### MEDICINE.

The Rearing of Children. A Practical Handbook for Mothers. By J. P. O'Hea, M.B., F.R.C.S. (The Scientific Press, Ltd., 29 Southampton Street, W.C. Price 2s. net.)

Dr. O'Hea's useful little book has been written in a lucid and terse manner, and in a style which, while it is plain and straightforward, is not without literary merit. All the chapters read "smoothly," and are not only instructive but interesting. This is a great desirability in a volume such as "The Rearing of Children," which primarily appeals to the lay public, and whose readers must have the attention wholly fixed before they can clearly understand the points brought before them. Dr. O'Hea has succeeded in winning such attention, and his book should prove popular. It is carefully and conscientiously written, and the practical instructions given are thorough and accurate. Necessarily the book is dogmatic; such books must always avoid the academic discussion of debatable subjects, and therefore the writer has done well in limiting himself to statements which have the general support of the experienced. A fair example of his method is seen in the chapter dealing with vaccination; it is a one-sided *résumé* of the question, with which an anti-vaccinationist will find much fault, no doubt, but it is a fair statement of the case for vaccination from the point of view of one who honestly believes in the efficacy of the Jennerian method, and as such it is valuable and instructive. We have paid particular attention to the simple prescriptions given in the book; they are excellent in every way. The chapters on rickets, constipation, and infectious diseases in childhood are all very good, and nowhere err on the side of excessive detail or skimpy descriptions. We are glad that the author lays stress on the importance of mouth-breathing exercises in cases of adenoids; our only adverse comment on his directions is that it is unnecessary and bad to make the children turn their toes out, soldier fashion, when they do these exercises. The little book is a simple but exceedingly useful text-book, which every mother and everyone who has the care of children should acquire.

Insanity in Everyday Practice. By E. G. Younger, M.R.C.P. Second edition, revised and enlarged. (London: Baillière, Tindall and Cox. 1910. Pp. 124. Price 3s. 6d. net.)

This is a reissue of a book which has already proved very useful to general practitioners in dealing with the harassing problems always confronting them when face to face with cases of insanity. It aims throughout at describing in a simple way the broad outlines of the subject, and in this it has certainly attained the object aimed at. In the space at disposal there is of course no room for a complete reference-book of insanity, but merely for classification of the main types and a general description of each of them. The chapters on certification, the examination of suspected lunatics, and the medico-legal aspects of insanity generally are excellent. It is essential that the general practitioner, who as a rule comes across insanity occasionally only, should have on his shelves some guide to the intricacies of this specialty; for it is one into whose mysteries he receives but little initiation as a student, nor does he as resident medical officer of a hospital gain any great experience how to deal with mental cases. To those who feel this need, and desire to fill it with an inexpensive yet trustworthy guide to the elementary principles on which are based the diagnosis, prognosis, and treatment of the insane, this volume can be cordially recommended.

Variations in Susceptibility of Guinea-pigs to Diphtheria Toxin. By H. J. Südmersen, Ph.D., and A. T. Glenny, B.Sc. Reprinted from the *Journal of Hygiene*, Vol. IX., No. 4, 1910. (From the Wellcome Physiological Research Laboratories, Herne Hill, London, S.E. Pamphlet of 10 pages.)

This interesting communication is a *résumé* of observations made for the past two years on the transmission of immunity to diphtheria toxin in guinea-pigs, during the course of which investigations it was observed that a seasonal variation in the dose is necessary in order to cause the death of control animals, a larger dose being necessary in summer than in winter. Again, it was found that in winter a guinea-pig takes longer to come up to the standard weight of 250 grams than in summer, and that the lethal dose varies directly with its age. The toxin selected was a stable one, prepared in December 1900. As the outcome of experiments suitably controlled, results have accumulated from more than 100 normal guinea-pigs, and although these cannot be taken as giving exact quantitative differences between minimal lethal doses they serve as indications of the extent of the differences. To obtain more accurate determinations it would be necessary to submit long series of guinea-pigs to injections of the toxin and definitely fix the lethal dose for each season of the year. The authors are at present carrying out this work. They conclude that the results they have reported in this pamphlet show that it is important, when dealing with the fatal dose of a toxin, or with any conclusion drawn from the loss in weight of an animal, to take into consideration the time of year when the experiment was performed.

An Index of Symptoms. By Ralph Winnington Leftwich, M.D. Fourth Edition. (London: Smith, Elder and Co. 1910. Pp. 451. Price 7s. 6d. net.)

This book, which has now reached a fourth edition, is one for the practising doctor rather than for the student, or at least for the examinee. Speaking generally, condensations are usually compiled for examination purposes, and, in so far as they are essentially devices for "cramming" and unpractical both in intention and effect, they are bad. But this manual, though condensation is carried in it to a fine art, is entirely free from this objection; and as a pocket companion, a reminder of things which, though they may be forgotten, are thoroughly understood, its value has been proved by the exhaustion of the three preceding editions. Dr. Leftwich's book needs therefore now no prolonged encomium in introducing it to the profession; it has already achieved its own introduction. In it the usual text-book principle of describing the symptoms that may be found in any one disease is reversed; the diseases are tabulated in which any one symptom may be found. The indexing, in fact, as the name of the book implies, is done under a semeiological instead of a pathological classification. Since the work is, in our view, adapted only for the wants of those whose purely academic career lies behind them, and who have had a good deal of practical experience, it is matter for regret that Dr. Leftwich has thought it necessary in this edition to add explanations, definitions, and sections upon diagnostic methods for the benefit of junior students. Apart from this one point, the book is more complete and useful than ever, for a good deal of new matter has been added since the last edition. In the concluding section, which is a list of rare diseases only to be diagnosed when the evidence is past dispute, are to be found several conditions which are in fact not very rare, though some of them are not uncommonly overlooked. Next time the author is called upon for a new edition he might reconsider carefully the claims of some of these diseases to be expunged from that section. One other recommendation must be disagreed with, and that is the advice to use three or four strokes of the plessor fingers on to the pleximeter finger; the student may require this number, but the trained physician should educate his faculties to make one stroke, or at the most two, suffice. With these trivial exceptions the book may be unreservedly commended as an experiment the success of which was long ago proved. Symptoms, as the author points out, are taken in the broadest possible sense, and include every factor in the diagnosis of disease.

The Action of an Active Principle from Apocynum. By H. H. Dale and P. P. Laidlaw. (Reprinted from *Heart*, November 1909. (From the Laboratories of Guy's Hospital and the Wellcome Physiological Laboratories, Herne Hill, S.E. Pamphlet of 29 pages.)

Apocynum has for some years enjoyed a varying reputation as an alternative to digitalis. In 1909 Finnemore isolated from Apocynum cannabinum a crystalline bitter principle, cynotoxin ($C_{20}H_{28}O_6$), which Laidlaw found to possess the characteristic action of apocynum. Almost simultaneously C. W. Moore isolated from Apocynum androsœmifolium a bitter principle, apocynamarin ($C_{28}H_{36}O_6.2H_2O$), which Dale ascertained to be the essential active principle. The authors of the present pamphlet, working together and comparing their results, come to the following conclusions: The characteristic effects of the two species of apocynum under consideration are due to the bitter principles named respectively cynotoxin and apocynamarin; there is no pharmacological indication of any difference between the two; the action is in all respects a characteristic digitalis effect, but careful investigation with the drug is still necessary before its place in the digitalis series can be established. Its vaso-constrictor action is much more powerful than that of strophanthin, whilst its action on the heart is but little weaker. Its diuretic action is similar to that of other drugs of the series. It is excreted or destroyed rapidly, and it would seem to be non-cumulative in its action. The authors think that this last fact will tell in its favour in practical therapeutics, since the pure active principle is free from the drawbacks of the crude drug. No irritative effects have been noticed either with oral or with subcutaneous administration of the pure principle, but the rapidity of its action enjoins caution with regard to subcutaneous dosage. On the other hand, experiments with crystalline apocynin (acetovanillone) show that this drug has but the feeblest physiological action.

### SURGERY.

Four Common Surgical Operations in India. By Major P. C. Gabbett, I.M.S. (London: Luzac and Co. Madras: Higginbotham and Co. 1910. Pp. 57. Price 1½ rupee.)

In his preface Major Gabbett pleads for a recognition of tropical surgery on similar lines to that universally extended to tropical medicine. There is, he says, no text-book on modern tropical surgery, although the conditions of surgical practice differ very considerably from those of temperate climates. The class of work and the class of patient are very different, as well as the temperature and the nature of the assistance available. In addition to this, certain operations, as those for elephantiasis and for tropical abscess of the liver, are almost peculiar to tropical surgery; whereas adenoids, appendicitis, gall stones, and senile prostates rarely require surgical intervention in the East. He deals first with hernia, and devotes much more space to the preliminary arrangements and pre-operative treatment than to the details of the operation itself. It is, of course, minute care on the numberless small points in the preparation of the instruments, ligatures, and patient that chiefly determine success or failure. The only noteworthy detail in technique is the adoption of a silver or aluminium-bronze filigree instead of the usual Bassini operation. Of hydrocele the author remarks that at the General Hospital, Madras, the surgeons see more cases than anywhere else in the world. He is strongly in favour of eversion as against excision, and estimates the recurrences after this simple and safe operation as no more than 2 or 3 per cent. Firm bandaging against the thigh is recommended for the first day or two. For hæmatocele, which is also much commoner in India than in Europe, a variety of procedure is adopted. Tapping, excision, and incision with packing are all useful in different cases; and no one method is to be rigidly adhered to for every hæmaotcele. In old men with large and long-standing swellings castration is the ideal treatment; but many refuse to submit to it. On elephantiasis Major Gabbett has much to say; too much, in fact, to be recapitulated here. He has come to the conclusion that the extensive enucleating operations on the leg, followed by skin grafting of the whole surface from the knee to the root of the toes, which were tried by several surgeons four or five years ago, are unsatisfactory in their final results, and unjustifiable. Lymphangioplasty has also not proved successful. Finally, are some excellent pages on cataract extraction by Major Herbert, whose forceful and practical hints on this subject will be extremely valuable to those who are about to commence a career of operative ophthalmic surgery in India, or indeed anywhere else.

Diseases of the Colon and their Surgical Treatment. By P. Lockhart Mummery, F.R.C.S. (Bristol: John Wright and Sons, Ltd. 10s. net.)

This book is an extension of the Jacksonian essay for 1909 on the Surgical Diseases of the Colon, and is an excellent exposition of a subject which is usually not well handled in the ordinary text-books. Starting with the surgical anatomy and embryology of the colon, the author proceeds to deal with the general diagnosis of colic diseases. In the first few chapters there is not indeed much that is original, although Mr. Mummery's remarks on the sensory nerves, from experience derived from operations, is interesting as confirming the general finding of other authorities that the normal mucosa of the large intestine possesses no tactile sensation, and that damage to the colon itself does not cause pain directly. Use has been made of the recent English investigations on the normal peristaltic movements of the colon, although not of the much more extended observations on the same subject by recent Continental investigators, whose results appear to us to be more in accordance with clinical evidence. The chapter on sigmoidoscopy is full and accurate, and we are glad to note that the author lays stress on the necessity for gentle handling when this useful aid to diagnosis is employed. When we come to the special diseases, Mr. Mummery's experience enables him to give many useful hints, and what is perhaps equally valuable, to deliver an opinion, which, in consideration of that experience, is instructive with regard to the relative merits of various forms of treatment. In cases of mucous colotis, where operative interference is necessary, he recommends appendicostomy, but is careful at the same time to give an impartial account of the results that may be expected from ileo-sigmoidostomy and—an operation which is much favoured by Continental authorities—cæcostomy. We are glad to see that he has thought it worth while to devote a chapter to that relatively common clinical entity pericolitis; but this chapter would have been more valuable to the general practitioner if more stress had been laid on the differential diagnosis between cases of appendicitis and right-sided pericolitis. The treatment of chronic constipation is fully and very ably discussed, and practitioners will find this probably the most practically useful of all the sections in the book, especially since the indications for operative treatment are clearly considered. That on malignant disease is equally full, and contains much interesting information. A few rarer conditions are dealt with, and considerable attention is paid to Hirshsprung's disease. The book is one of the best monographs on the diseases of the colon with which we are acquainted, and should be a useful addition to the practitioner's bookshelf.

### TROPICAL MEDICINE.

Lessons on Elementary Hygiene and Sanitation, with Special Reference to the Tropics. By W. T. Prout, C.M.G., M.B., C.M.Edin. (London: J. and A. Churchill. 2s. 6d. net.)

This book embodies a series of lectures which the author has added to and brought out in the present form. It is a very admirable little text-book, and especially is it adapted for use in the Schools of Tropical Medicine. It is well written and easy to understand, the type is good, and the many illustrations are excellent. It is undoubtedly a book that will prove of great service to the student preparing for an examination in State medicine, or wishing to make a special study of tropical hygiene.

A Guide to the Preservation of Health in West Africa. By Henry Strachan, C.M.G., L.R.C.P. Lond., M.R.C.S. Eng., Principal Medical Officer of Southern Nigeria. (London: Constable and Co., Limited. 1910. Pamphlet of 23 pages. Price 6d. net.)

This little book, based on a pamphlet written for the Government of Southern Nigeria for distribution to Government officials, is intended mainly for the use of Europeans whose work necessitates their taking up their abode in Nigeria. Advice is given on anti-malarial precautions, the preparation of drinking water, protection from the heat of the sun, sanitation, clothing, and the taking of medicines such as quinine and purgatives. The author lays stress on the value of a 39 per cent. of formaldehyde in the treatment and prevention of boils, a disease which is very prevalent in Nigeria. One drop applied with a brush or at the end of a match to the boil, and repeated if necessary two or three times in the course of forty-eight hours, will speedily effect a cure. Plans of a mosquito-proof house and of the proper apparatus necessary for the collection of rain-water are included in the work and increase its value.

Health, Progress, and Administration in the West Indies. By Sir Rubert W. Boyce, M.B., F.R.S. (London: John Murray. 10s. 6d. net.)

In March 1909 Sir R. W. Boyce was requested by the Secretary of State for the Colonies to visit Barbados in order to investigate an epidemic of yellow fever then raging in the Colony. While there he made use of the opportunity to visit the Windward Islands, Trinidad, and British Guiana, and to report upon the health conditions of these places. The result of these travels is embodied in this book, which from cover to cover abounds in interesting detail. It gives an admirable *résumé* of the hygienic conditions of the West Indies. No pains were spared by those with whom Sir Rubert Boyce was brought into contact to afford him the fullest opportunity to make a thorough inquiry into the sanitation and the modes of living on these islands. The opening chapters are devoted entirely to the study of the history, mode of transmission, and nature of yellow fever. For this treatise on the deadly disease the book, apart from the other useful information it contains, is most valuable, and the author is to be congratulated upon the excellence of his detailed account of this plague. The first record of yellow fever seems to have been in the year 1493, when it broke out in the City Isabella, in St. Domingo, at the time of its foundation by Columbus. Subsequent outbreaks are recorded at Capara in Porto Rico in 1508, and at St. Pierre in Martinique in 1735. It appears to have started at the foundation of these cities, and it is interesting to note this, because in the earliest account that we have of the disease, Père Du Tertre (1635) relates in his "Histoire Générale des Antilles" "how it was known as *coup de barre*, owing to the intense muscular pains which are characteristic of the disease, and how it attacked those clearing the land and exposed to so-called poisonous vapours and exhalations."

Yellow fever appears to have been known by many names. In 1649 Père Labat, who landed in Martinique, describes it, and calls it *mal de Siam*, "because it was thought it was imported from Siam in the ship *Oriflamme*." It was known also as "Bulam fever," and in Barbados in 1691 it went by the name of Kendal's disease. Yellow fever was not unknown in epidemic form in Europe, and even penetrated to France and England. The author regards the fever as a "dying disease." That the gradual stamping out of yellow fever in the West Indies is very greatly due to the advance made in sanitation Sir Rubert proves beyond doubt. In the eighteenth century everyone—man, woman, and child—who landed in the West Indies seems to have had to run the gauntlet of this abomination. In 1741 in the siege of Carthagena, out of a total of 12,000 men, 8,431 lost their lives through fever—an appaling mortality! Nor did the country in those days seem only to have yellow fever to contend against. Polluted well water produced cholera, while *mal d'estomac*, typhoid, and dysentery occurred in epidemics. There appeared to be no system of drainage at all, and the hovels in which the inhabitants dwelt were overcrowded, evil smelling, and drainless; fever was ever present, and only too often smallpox, leprosy, tetanus, and *mal rouge* raged. The author devotes a chapter on the old and new theories of the transmission of the disease. He tells us that the fever is "caused by a specific virus, the nature of which is as yet undiscovered," but which is transmitted only by the *stegomyia calopus*." An extraordinary amount of work has been expended upon this subject, and the ætiology, symptoms, and treatment have received the closest attention. Now that the data for prophylaxis are sufficient and precise, they ought to yield unerring results. Time and space will not permit us to go further into this absorbingly interesting book, but to medical men and laymen alike, and to anyone interested in the health conditions of the present day, it is a work that will well repay the reading.

### MISCELLANEOUS.

The History of St. George's Hospital. By G. C. Peachey, M.R.C.S., L.R.C.P. Part I. (London: John Bale, Sons, and Danielsson. 1910. Price 2s. 6d. net.)

The first part of "The History of St. George's Hospital," to whose approaching issue attention was directed on May 28, has now been published. After a few pages in which the origin and growth of charitable institutions for the relief of the sick in England are discussed and described, the author plunges at once into the historic quarrel which led to the secession of the staff of the Westminster Infirmary (as it then was) and the acquisition of Lanesborough House at Hyde Park Corner. This was in 1733, and was, in fact, the foundation of St. George's Hospital. In dealing with the incidents which led to the schism, contemporary documents are freely quoted, often in their entirety, and both sides of this almost forgotten dispute are presented fully and carefully in the pamphlets and broadsheets of their supporters. The page is large and excellently printed in clear type, while the cover is of green; perhaps Mr. Peachey will be able to explain in a subsequent number how green came to be the chosen colour of the hospital. In the second part are promised a description of Lanesborough House and its conversion into St. George's Hospital, with an account of Hyde Park Corner and its surroundings at that date with contemporary maps and views.

City of London Year-book and Civic Directory for 1910. W. H. and L. Collingridge. (City Press Office, Aldersgate Street, E.C. 5s. net.)

This book replaces the old City of London Directory, which was in existence for thirty-nine years. The successor is in every way worthy of the support which was extended to the old compilation, for it is an admirable handbook of reference so far as City matters are concerned. The lists of liverymen and livery companies are up to date and accurate, and a feature of the work is the series of short biographies of prominent City officials which makes the book a practically useful "Who's Who?" so far as the capital of the Empire is concerned. Much interesting information is given with regard to schools and hospitals. We note under the heading "Classified List of Selected Trades" two addresses of medical men who, so far as we are aware, have no connection with the City. One is described as a surgeon-aurist and the other as a surgeon-oculist. We would suggest to these gentlemen that such advertisement is worth the attention of the General Medical Council.

History of the Human Body. By Harris Hawthorne Wilder. (New York: Holt; London: Bell. Pp. 550, with numerous diagrams. Price 14s. net.)

Professor Wilder prefaces his book by the statement that it is written specially with regard to the needs of the medical student, and does not therefore discuss questions of comparative anatomy which have little bearing on the human body. We cannot, however, help feeling that with the best intentions in the world, Professor Wilder has hardly succeeded in his aim. Such a course of zoology as he proposes would probably require three years' laborious work in the dissecting laboratory, besides a vast amount of extraneous reading, for it is scarcely likely that a student would take the various problems without going to the original work on the subject. The book is too advanced for the ordinary medical student, and too sketchy for the advanced zoologist. Controversial matter is unnecessarily included, and we fail to see the value from any practical point of view of a discussion either of the phylogeny of the vertebrates or of their ancestry. In fact, the book would have been far better if it had taken man and traced the devolution of his various parts, thus including a paper on this subject in the second and not in the first examination for the M.B. degree. That far too little biology is now studied by the medical student is probably true; but Professor Wilder does not really tackle this problem satisfactorily. The genealogical trees given in the second chapter are all very well for a lecture-room where necessary explanations can be given, but their inclusion in the book seems likely to lead to great error, particularly when we remember that nothing is certain about the line of descent of man. The diagrams are numerous, and in many cases well chosen, but there is room for much improvement in this direction, and the inclusion of some really good original drawings would have been most satisfactory. On the whole, if we cannot award unstinted praise to the execution of Professor Wilder's idea, we can at least say that the idea itself deserves serious consideration, and that a perusal of his work will show how necessary a zoological basis is for anatomical and physiological superstructures.

